# Resveratrol Alleviates Corticosterone-Induced Hepatic Lipid Metabolism Disorder and Oxidative Stress by Regulating the Nrf2 and AMPK/Sirt1 Signaling Pathways in AA Broilers

**DOI:** 10.3390/ani16111574

**Published:** 2026-05-22

**Authors:** Chendi Fu, Jiawei Ma, Xiaoxuan Zong, Jin Sun, Xingjun Feng

**Affiliations:** College of Animal Science and Technology, Northeast Agricultural University, Harbin 150030, China; 13065053569@163.com (C.F.); ma1014808476@163.com (J.M.); 15004503775@163.com (X.Z.); sunjing1228228@163.com (J.S.)

**Keywords:** broiler, resveratrol, stress, corticosterone, liver, lipid metabolism disorder

## Abstract

Excessive glucocorticoids induced by stress trigger hepatic lipid metabolism disorder and oxidative stress in poultry, impairing growth performance and welfare. At the same time, resveratrol (RSV) has antioxidant and lipid-regulating properties, but the protective mechanisms in corticosterone (CORT)-challenged broilers remain unclear. This study investigated RSV’s effects on CORT-induced hepatic damage in AA broilers, with 240 one-day-old broilers randomized into three groups: control (basal diet), CORT (basal diet + 4 mg/kg BW CORT intraperitoneal injection), and RSV (400 mg/kg RSV-supplemented diet + CORT injection). Growth performance, hepatic redox status, serum biochemistry, liver histopathology, and gene/protein expression related to antioxidant/lipid metabolism were determined. The growth performance of AA broilers injected with CORT was significantly affected, showing reduced body weight gain (*p* < 0.05), increased abdominal fat content (*p* < 0.05), and hepatomegaly (*p* < 0.05). The addition of RSV in the diet significantly reduced abdominal fat accumulation and hepatomegaly (*p* < 0.05), improving the growth performance of broilers; Effects of RSV on liver function and lipid metabolism of CORT-treated AA broilers: After CORT injection, serum alanine aminotransferase (ALT), aspartate aminotransferase (AST), alkaline phosphatase (ALP) activity and total bile acid (TBA) content significantly increased (*p* < 0.05). Hepatic total cholesterol (TC) and triglycerides (TG) increased after CORT injection (*p* < 0.05), causing severe liver damage. RSV supplementation could reverse the increases in serum ALP, ALT, and AST activity (*p* < 0.05) and reduce TBA content in stressed broilers (*p* < 0.05). TC and TG levels in the liver decreased under the alleviation of RSV (*p* < 0.05), and serum TG levels declined (*p* < 0.05). Microscopic and ultrastructural observations showed that after CORT injection, hepatic tissue cells were swollen, scattered fat vacuoles were present, pores were enlarged, and intracellular lipid droplets appeared. The RSV group significantly alleviated hepatocyte damage, reduced vacuolation, showed uniform chromatin, and decreased lipid droplets. RSV significantly mitigated the CORT-induced increase in SREBP-1 mRNA and protein expression and the decrease in PPARα protein expression; CORT caused a decline in the antioxidant function of AA broiler livers, with significant decreases in SOD and GSH-PX (*p* < 0.05), and the expression of Nrf2 and its downstream genes also showed a decreasing trend. Compared to the CORT group, the RSV group exhibited significant increases in liver CAT, SOD, and GSH-PX (*p* < 0.05), and Nrf2 protein expression was elevated (*p* < 0.05). In summary, resveratrol can alleviate the decline in growth performance, liver steatosis, and hepatic oxidative stress in AA broilers induced by CORT, downregulate lipogenic genes such as SREBP-1c, regulate liver lipid metabolism, and mitigate CORT-induced hepatic oxidative stress in broilers by upregulating the Nrf2 pathway.

## Simple Summary

Resveratrol is a natural polyphenolic compound widely distributed in nature, with biological functions including antioxidant activity, enhanced animal growth performance, and regulation of liver lipid metabolism. However, there are currently few comparative studies on the alleviating effects of resveratrol on stress-induced lipid metabolism disorders and oxidative stress in broilers and the corresponding mechanisms. Resveratrol can mitigate the decline in growth performance, hepatic steatosis, and hepatic oxidative stress induced by corticosterone treatment in AA broilers. In this article, resveratrol can alleviate liver steatosis and hepatic oxidative stress in AA broilers induced by CORT treatment, downregulate lipogenic genes such as SREBP-1c (Sterol Regulatory Element-Binding Protein-1c), regulate hepatic lipid metabolism, and activate Nrf2 (Nuclear factor erythroid 2-related factor 2) and modulate the AMPK/Sirt1–SREBP-1c/PPARα axis, protecting AA broilers from CORT-induced liver lipid metabolism disorders and oxidative stress, providing a theoretical basis for RSV as a natural feed additive to mitigate stress-related metabolic diseases.

## 1. Introduction

Stress is a systemic, non-specific response to a variety of environmental, social, and psychological stimuli that challenge the body’s homeostasis. While stress is a natural defense mechanism, excessive stress, termed “inferior stress”, can lead to significant discomfort, pathological changes, and even life-threatening conditions. Poultry are particularly susceptible to diverse stressors throughout their production cycle, such as transportation stress and temperature fluctuations, which can disrupt their physiological balance [1]. Prolonged exposure to stressors can lead to physiological stress, which, in turn, can negatively impact production performance by altering various bodily functions. For instance, a reduction in feed intake can impede the growth and development of poultry, increase the feed-to-meat ratio, and decrease meat and egg quality and production rates [2]. Physiological stress has also been shown to upregulate hepatic lipid synthesis genes and promote liver lipid deposition in poultry [3]. Additionally, stress can compromise poultry immunity, predispose them to diseases, and even result in mortality.

The stress response is a dynamic process that maintains physiological homeostasis by activating the sympathetic nervous system and the hypothalamic–pituitary–adrenal (HPA) axis, which involves a cascade of endocrine adjustments. Corticotropin-releasing hormone (CRH) stimulates the secretion of adrenocorticotropic hormone (ACTH) from the anterior pituitary, which in turn promotes the secretion of glucocorticoids (GCs) by the adrenal cortex [2]. GCs, primarily cortisol and corticosterone (CORT) in mammals and predominantly CORT in poultry, are the terminal regulators of the HPA axis and play a crucial role in the body’s stress response [4]. A high level of CORT can exacerbate fear in animals, thereby increasing stress. CORT injections have been shown to induce stress responses in broilers, adversely affecting BW, BWG, and feed conversion rates [5]. Excessive GCs exposure has been linked to metabolic syndromes in both mammals and birds, including non-alcoholic fatty liver disease (NAFLD), a hepatic manifestation of metabolic syndrome that can progress to liver failure. Although the molecular mechanisms of NAFLD are not fully understood, the role of hepatic lipid metabolism is widely recognized [6,7]. Exogenous CORT treatment can lead to abnormal liver fat deposition [8,9], where impaired lipoprotein transport results in fat accumulation in hepatocytes, potentially causing a fatty liver accompanied by mitochondrial dysfunction, lipid peroxidation, and inflammation, thereby increasing the liver’s vulnerability to damage [10]. Obesity-related insulin resistance and hepatic oxidative stress are also considered significant factors in the development of hepatic steatosis and liver injury, marking the early stages of NAFLD and potentially persisting throughout disease progression [11,12].

Resveratrol (RSV), a natural stilbene found primarily in red wine, grapes, rhubarb, and blueberries, possesses a range of biological benefits, including anti-inflammatory and anti-cancer properties, and the regulation of lipid and glucose metabolism [13]. Recent studies have highlighted RSV’s therapeutic potential in mitigating oxidative stress and lipid metabolism disorders [14,15], and it is a more potent inhibitor of hepatic oxidative stress and liver injury, demonstrating a stronger hepatoprotective effect in high-fat-fed rats by reducing hepatic oxidative stress [16,17]. Despite these promising findings, there remains a critical gap in the existing research: limited data are available regarding RSV’s protective effects against insulin resistance and hepatic oxidative stress induced by stress-related glucocorticoid excess, and its specific regulatory role in hepatic lipid metabolism under such stress conditions remains unclear. Given that stress is a major contributor to hepatic lipid metabolism disorders and NAFLD-like lesions in poultry, which severely impacts poultry production and welfare, filling this research gap is of great practical and academic significance. Therefore, this study aims to evaluate the in vivo protective potential of RSV on hepatic redox status and lipid metabolism, using an AA broiler chicken model of lipid metabolism disorder induced by CORT injection. The findings of this study are expected to provide theoretical support for the application of RSV in alleviating stress-induced hepatic damage in poultry and offer new insights into the prevention and control of stress-related metabolic diseases in avian species.

## 2. Materials and Methods

### 2.1. Biochemical Reagents

High-purity RSV (>98%) was purchased from Jing Zhu Biotechnology Co., Ltd. (Nanjing, China). CORT was purchased from Sigma-Aldrich Corp. (St. Louis, MO, USA).

### 2.2. Animals Grouping and Management

A total of 240 1-day-old AA broilers were randomly divided into three groups of eight replicates per group with 10 birds per replicate: (1) Control group (CON), broilers fed a basal diet; (2) CORT group: broilers fed a basal diet, subjected to CORT injections from 35 days of age; (3) RSV group, broilers a diet fortified with 400 mg/kg RSV and challenged with CORT from 35 days of age. The RSV dosages were selected based on previous studies [18,19]. The basal diet complied with the nutritional guidelines of the National Research Council (NRC, 1994) for the starter (1–21 days) and growth (22–42 days) stages. Diet composition and nutrient levels are detailed in the Supplementary Materials (Supplementary Table S1) [Zhang]. Broilers had ad libitum access to feed and water from 1 to 41 days of age. From days 35 to 41, the CORT and RSV groups received 4 mg/kg BW CORT via intraperitoneal injection twice daily, while the CON group received an equivalent volume of saline. Body weight and feed intake were recorded at days 35 and 41. All animal care and experimental protocols adhered to the National Research Council Guidelines (1996) [20] and were approved by the Ethics and Animal Welfare Committee of Heilongjiang Province, China (revised 2016) [21]. The study was approved by the Institutional Animal Care and Use Committee of Northeastern Agricultural University (NEAUEC20220248).

### 2.3. Sample Collection

Samples were collected at 9:00 a.m. When collecting blood and tissue samples, one broiler from each replicate group with the closest average weight was selected, with samples collected from a total of 8 birds per group. Blood was collected from the wing veins of the broilers. Blood samples were incubated at 37 °C for 30 min, then centrifuged at 3500 rpm at 4 °C for 15 min to separate the serum. The serum was subsequently aspirated and stored at −80 °C for subsequent testing. Following blood collection, the broilers were euthanised. Following euthanasia, the abdominal cavity was opened, and the bilateral symmetrical yellow adipose tissue located in the lower posterior abdomen, anterior to the cloaca and medial to the femur, was rapidly removed and weighed. At the same time, liver tissue samples were rapidly removed and weighed. Liver tissue was rinsed in ice-cold phosphate-buffered saline (PBS, pH 7.2–7.4), and approximately 1 cm^2^ samples were fixed in 4% paraformaldehyde for histological examination. A separate 1 mm^3^ portion was immersed in 2.5% glutaraldehyde phosphate buffer (pH 7.2) for electron microscopy. The remaining liver tissue was snap-frozen in liquid nitrogen and stored at −80 °C for subsequent biochemical analyses.

### 2.4. Biochemical Analysis

Serum biochemical markers of liver injury and lipid metabolism, including alanine aminotransferase (ALT), aspartate aminotransferase (AST), alkaline phosphatase (ALP), total triglycerides (TG), and total cholesterol (TC), were quantified using an automated biochemical analyzer (Roche Cobas Mira Plus, Basel, Switzerland) and commercial diagnostic kits (Jiancheng Biotech, Nanjing, China). Hormonal levels pertinent to lipid metabolism, such as thyroid hormones (total T3, total T4), insulin (INS), CORT, adiponectin (ADP), and leptin (LEP), were determined using ELISA kits (mlbio, Shanghai, China) according to the manufacturer’s guidelines. Additionally, blood glucose (Glu) levels were evaluated using ELISA kits (mlBio, Shanghai, China) to assess insulin resistance in broilers.

### 2.5. Oil Red O Staining Analysis of Liver Tissue

Liver tissues fixed in 4% paraformaldehyde were sequentially dehydrated in 20% and 30% sucrose solutions at 4 °C. The tissues were then trimmed flat and embedded in OCT compound. After precooling in a cryostat, the tissues were sectioned into 8–10 μm slices, mounted on slides to air-dry, and stored at −70 °C. The sections were sequentially placed in staining baths containing distilled water, 60% isopropanol solution, and Oil Red O working solution for 2 min, 2 min, and 5 min, respectively, followed by differentiation in 60% isopropanol, washed in distilled water for 2 min, immersed in haematoxylin for 1 min, rinsed in tap water for bluing, and finally mounted with glycerol gelatin. Prepared liver tissue sections were observed and images captured using an Olympus BX53 microscope(Olympus Corporation, Tokyo, Japan).

### 2.6. Histopathological Observation of the Liver

The preparation of pathological tissue sections and scanning electron microscopy samples of the liver refers to the method described by Zhang et al. [22]. Liver samples fixed in paraformaldehyde were dehydrated and embedded in paraffin wax. Sections of approximately 5 μm were cut from the paraffin blocks and stained with hematoxylin and eosin (H&E) for microscopic examination using a Nikon Eclipse Ci-L light microscope (Nikon, Tokyo, Japan). For ultrastructural analysis, liver samples were dehydrated in 2.5% glutaraldehyde and postfixed in 1% osmium acid before embedding in resin. Ultrathin sections were then stained with uranyl acetate and lead citrate and examined using a Hitachi H-7650 transmission electron microscope (HITACHI, Tokyo, Japan).

### 2.7. Measurement of Antioxidant Index in Liver Tissues

Liver tissues were homogenised in ice-cold 0.85% saline solution, and a 10% homogenate was prepared using a LAWSON-24 high-speed grinder (LAWSON, Beijing, China) at 4 °C. The total protein concentration of the samples was determined using the BCA protein concentration assay kit from Nanjing Jiancheng Biotechnology Research Institute (Nanjing, China); all experimental procedures were carried out strictly in accordance with the kit instructions. Based on the measured protein concentrations, the protein samples were diluted to a uniform concentration, followed by the determination of antioxidant enzyme activities (including total antioxidant capacity (T-AOC), superoxide dismutase (SOD), glutathione peroxidase (GSH-Px) and catalase (CAT)), as well as malondialdehyde (MDA) levels, were all measured using assay kits from Nanjing Jiancheng Biological Engineering Research Institute (Nanjing, China) and a UV-Vis spectrophotometer (UV1100, MAPADA, Shanghai, China).

### 2.8. Lipid Metabolism Testing in Liver

Hepatic homogenates were prepared similarly to the antioxidant index assay, using precooled 0.85% saline solution. After centrifugation, the supernatant was analyzed for TG and TC content using commercial assay kits from Nanjing Jiancheng Bioengineering Institute (Nanjing, China), following the provided protocols.

### 2.9. RNA Extraction and Real-Time Quantitative PCR Analysis

Total RNA was extracted from 100 mg aliquots of liver tissue preserved at −80 °C using TRIzol reagent according to the manufacturer’s guidelines (TaKaRa, Tokyo, Japan). RNA quality and purity were assessed by the A260/A280 ratio, with values between 1.8 and 2.0 indicating acceptable integrity, using a spectrophotometer (Implen Nanophotometer P-330, Munich, Germany). cDNA was synthesized from 1 μg of RNA per liver sample using the Prime Script™ RT kit and gDNA Eraser (TaKaRa, Dalian, China) according to the manufacturer’s instructions. Quantitative real-time PCR (qRT-PCR) was conducted on a Quantagene q225 system (Kubo Tech, Beijing, China) with SYBR Green detection (TaKaRa, Tokyo, Japan). Relative gene expression was determined using the 2^−ΔΔCt^ method, with β-actin serving as the endogenous control. Primer sequences for the target genes of the broiler, sourced from NCBI and synthesized by Sangon Biotech Co. (Shanghai, China), are detailed in Supplementary Table S2.

### 2.10. Protein Extraction and Protein Blotting Assay

Liver tissue was homogenized in RIPA buffer containing 1 mM PMSF (Beyotime Biotechnology, Shanghai, China), and protein concentration was quantified using a BCA assay kit (Beyotime Biotechnology). Proteins were denatured by boiling after mixing with the loading buffer. Following separation by 12% SDS-PAGE, proteins were transferred onto polyvinylidene difluoride (PVDF) membranes. Membranes were blocked with 5% skim milk in TBST at 37 °C for 2 h, then incubated with primary antibodies (GAPDH (glyceraldehyde-3-phosphate dehydrogenase); Nrf2 (nuclear factor erythroid 2-related factor 2); SREBP-1c (sterol regulatory element-binding protein-1c); PPARα (peroxisome proliferator-activated receptor α); AMPK (AMP-activated protein kinase); SIRT1 (silent information regulator 1); PI3K (phosphoinositide 3-kinase); AKT (protein kinase B); P-AKT (phosphorylated AKT). Beyotime Biotechnology) diluted 1:1000 overnight at 4 °C. After incubation with horseradish peroxidase-conjugated secondary antibodies (1:1000; Beyotime Biotechnology) for 2 h at 37 °C, protein bands were visualized using an ECL chemiluminescence kit (Beyotime Biotechnology) on a gel documentation system (UVItec, Cambridge, UK). Protein expression levels were quantified and normalized relative to GAPDH expression.

### 2.11. Statistical Analysis

Data from each sample were derived from six replicate measurements. Results are presented as mean ± standard error of the mean (SEM) and were analyzed using SPSS software (version 26.0, SPSS Inc., Chicago, IL, USA). The significance of differences among groups was determined using one-way ANOVA, followed by the LSD post hoc test. A *p*-value of less than 0.05 was considered to indicate statistical significance. Photographs were processed with Adobe Photoshop 2021CC (Adobe Systems, San Jose, CA, USA), and graphs with error bars representing standard deviation were generated using GraphPad Prism (version 9.0.0, GraphPad Software, San Diego, CA, USA).

## 3. Results

### 3.1. Growth Performance

As shown in Table 1, there were no significant differences in initial body weight and average daily feed intake among broilers in different treatment groups (*p* > 0.05). Meanwhile, the final body weight (Final BW) differed significantly among the treatment groups (*p* < 0.05). The differences in average daily gain (ADG), feed-to-gain ratio (F/G), abdominal fat index, and hepatic index among the treatment groups were extremely significant (*p* < 0.01). After the CORT challenge, the final body weight of the CORT group was significantly lower than that of the other groups (*p* < 0.05). Compared with the CORT group, the RSV group, although not reaching the level of the control group, showed a significant increase in final body weight (*p* < 0.05). Compared with the control group, the CORT and RSV groups had significantly reduced average daily gain (*p* < 0.01) and significantly increased F/G (*p* < 0.01). Injection of CORT significantly increased abdominal fat and hepatic indices in broilers (*p* < 0.05). In contrast, RSV intervention significantly reduced both indices (*p* < 0.05), particularly lowering the abdominal fat index to the level of the control group.

### 3.2. Hepatic Redox Status

Table 2 shows the redox status of the liver following cortisol treatment. In broiler chicken livers, the intergroup differences in all antioxidant parameters reached the level of extreme significance (*p* < 0.01). Post hoc tests indicated that, compared with the CON group, cortisol treatment significantly reduced total antioxidant capacity (T-AOC), superoxide dismutase (SOD), glutathione peroxidase (GSH-Px), and catalase (CAT) (*p* < 0.01), whilst significantly increasing malondialdehyde (MDA) levels (*p* < 0.01). RSV intervention significantly reversed the CORT-induced decreases in T-AOC (*p* < 0.01), CAT (*p* < 0.05), and GSH-Px (*p* < 0.01) activity, restoring CAT and GSH-Px activity to levels observed in the CON group (*p* > 0.05). Furthermore, compared with CORT induction, RSV treatment significantly reduced MDA levels (*p* < 0.05). However, the effect of RSV intervention on SOD activity showed no significant difference compared with the CORT group (*p* > 0.05).

### 3.3. Hepatic Pathology and Ultrastructural Assessment

**Figure 1:**
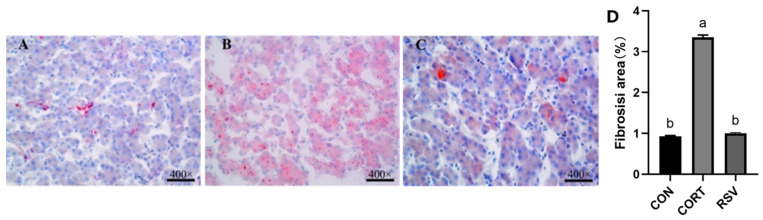
Effect of dietary RSV on oil red O staining of liver tissue of broilers challenged with CORT (400×). (**A**) CON group; (**B**) CORT group; (**C**) RSV group; (**D**) Quantification of Oil Red O-positive areas in liver sections. a, b The means with different letters in the superscript indicate significant differences (*p* < 0.05). No broilers died during the trial.

Oil Red O-stained histological sections revealed a pronounced enhancement in hepatic lipid deposition following CORT challenge. From Figure 1, it can be seen that the control group had only a small amount of red fat granules, the CORT injection group had a significantly increased amount of red fat granules (*p* < 0.01), and after RSV treatment, the red fat granules were significantly reduced (*p* < 0.01), with only a small amount of red remaining. This study used resveratrol to alleviate corticosterone-induced lipid metabolism disorders and oxidative stress in AA broiler livers, and Oil Red O staining was performed to observe lipid accumulation within liver cells.

**Figure 2:**
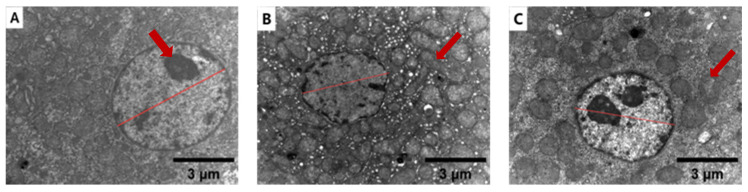
Effect of RSV on the liver ultrastructure of broilers challenged with CORT (scale bar = 3 μm). Nuclear maximum diameter: RVS: d = 4.907 μm; CORT: d = 4.272 μm; CON: d = 5.804 μm. (**A**) CON group; (**B**) CORT group; (**C**) RSV group. The arrow points to the cell nucleus; the red line indicates the nucleus’s maximum diameter.

In the ultrastructural examination of hepatocytes depicted in Figure 2, the cell nucleus is compressed by lipid droplets, causing a reduction in volume. These are indicative of severe liver tissue damage, thus reflecting the development of severe fatty liver in broilers. In stark contrast, the CON and RSV-treated groups exhibited preserved liver tissue morphology and integrity.

### 3.4. Hematology and Biochemistry

Serum aminotransferase activities and bile acid concentrations are pivotal biomarkers for liver injury. As shown in Table 3, in broiler livers, the differences in liver function parameters among groups were highly significant (*p* < 0.01). Post hoc tests indicated that, compared with the CON group, CORT treatment significantly increased ALP, ALT, AST, and TBA levels (*p* < 0.01). Meanwhile, ALP and TBA were significantly elevated in the RSV group (*p* < 0.01), with no significant difference in ALT (*p* > 0.05). RSV intervention significantly attenuated the CORT-induced increases in ALP, ALT, AST, and TBA (*p* < 0.05).

Table 4 shows that there were no significant differences in serum T4 levels among the different treatment groups (*p* > 0.05), while there were significant differences in serum T3/T4 levels among the different treatment groups (*p* < 0.05), and extremely significant differences in other hormone levels among the different treatment groups (*p* < 0.01). Compared with the CORT group, RSV treatment significantly increased T3 levels and the T4-to-T3 conversion rate (*p* < 0.05), with no significant difference compared with the CON group. Compared with the CON group, following CORT injection, serum ADP and LEP concentrations were significantly reduced (*p* < 0.05), while serum CORT and insulin (INS) levels were significantly increased (*p* < 0.01). However, after RSV treatment, serum CORT and INS levels were significantly lower compared with the CORT group (*p* < 0.01). Homeostasis model assessment of insulin resistance (HOMA-IR) indicated that CORT injection resulted in significant insulin resistance in broilers (*p* < 0.01), which was also confirmed by elevated blood glucose levels (*p* < 0.01). Compared with the CORT group, the addition of RSV significantly reduced HOMA-IR levels (*p* < 0.01).

### 3.5. Lipid Metabolism

The effects of adding RSV to the feed on lipid metabolism in broilers injected with CORT are shown in Table 5. Between different treatment groups, there were significant differences in low-density lipoprotein cholesterol (LDL-C) levels (*p* < 0.05) and extremely significant differences in other lipid metabolism function levels (*p* < 0.01). Compared with the CON group, the serum and liver TC and TG levels in the CORT group were significantly elevated (*p* < 0.01). In addition, compared with the CON group, the serum HDL-C concentration in the CORT group was significantly decreased (*p* < 0.05), while LDL-C concentration was extremely significantly increased (*p* < 0.01). RSV had no significant effect on serum TC and LDL-C levels in broilers after cort injection, but significantly reduced serum and liver TG levels (*p* < 0.01) and significantly increased HDL-C levels to the control group level (*p* < 0.05).

### 3.6. Hepatic Expression of Nrf2 and Downstream Target Genes

Table 6 shows that there were no significant differences in NQO1 mRNA levels between the different treatment groups (*p* > 0.05). In contrast, there were highly significant differences in mRNA levels of other antioxidant genes among the treatment groups (*p* < 0.01). Following cortisol treatment, the mRNA levels of Nrf2 (*p* < 0.01), HO-1 (*p* < 0.05), and GSH-Px (*p* < 0.05) showed a significant downward trend, whilst CAT and SOD also exhibited a downward trend; however, this change did not reach statistical significance (*p* > 0.05). In the RSV group, the mRNA levels of Nrf2 and HO-1 were extremely significantly higher than those in the control and cortisol groups (*p* < 0.01), whilst the mRNA levels of the CAT, SOD, and GSH-Px genes were also significantly elevated compared with the control group (*p* < 0.05) and extremely significantly elevated compared with the CORT group (*p* < 0.01). Corticosterone injection significantly downregulated Nrf2 and Sirt1 protein expression. Meanwhile, RSV intervention could significantly restore Nrf2 and Sirt1 and further activate AMPK, indicating that RSV may alleviate corticosterone-induced oxidative stress and metabolic disorders through activation of the Nrf2/Sirt1/AMPK signaling axis. 

**Figure 3:**
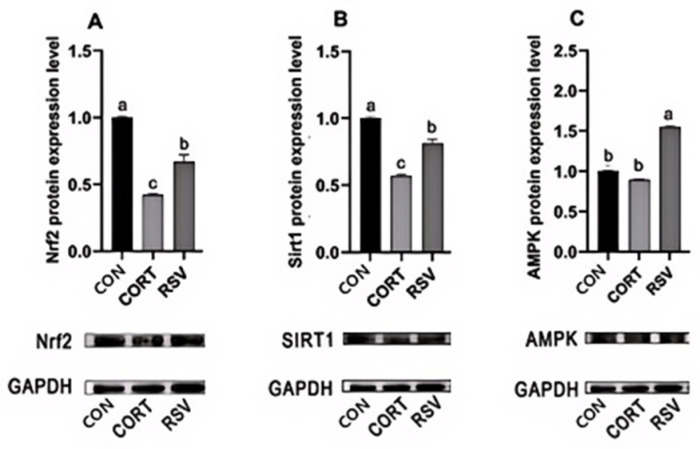
Effect of dietary RSV on the protein levels of Nrf2, Sirt1, and AMPK of broilers challenged with CORT. (**A**) Nrf2 protein; (**B**) Sirt1 protein; (**C**) AMPK protein. AMPK is total AMPK. Different letters in the same column with shoulder labels indicate significant differences (*p* < 0.05), and the same letters or no letters indicate non-significant differences (*p* > 0.05).

As shown in Figure 3, the protein levels of Nrf2 and Sirt1 in the CORT group were significantly reduced compared to the CON group (*p* < 0.05). In contrast, the RSV group showed significant upregulation in Nrf2, Sirt1, and AMPK protein levels compared with the CORT group (*p* < 0.05).

### 3.7. Expression of Lipid Metabolism Genes

The effect of RSV on the mRNA levels of seven lipid metabolism-related genes in the liver of broilers stimulated by CORT was determined by qRT-PCR. As shown in Table 7, there were significant differences in mRNA levels of lipid-synthesis genes among the different treatment groups (*p* < 0.01). Compared with the CON group, CORT significantly increased the mRNA levels of four genes related to lipid synthesis (SREBP1c, ACC, FAS, and PI3K) (*p* < 0.01). Meanwhile, the RSV group showed significant increases in SIRT1, SREBP1c, ACC, PPARα, and CYP7A1 levels (*p* < 0.01). RSV treatment significantly reduced the mRNA levels of FAS, ACC, and PI3K genes in the livers of broilers stimulated by CORT (*p* < 0.05). It significantly increased the mRNA levels of SIRT1, CYP7A1, and PPARα genes (*p* < 0.05). Corticosterone induces excessive liver lipid synthesis in broilers by upregulating the expression of genes related to hepatic lipid synthesis (SREBP-1c, ACC, FAS, and PI3K). In contrast, RSV intervention can effectively counteract corticosterone-induced hepatic lipid metabolism disorder by inhibiting the transcription of genes involved in lipid synthesis, such as ACC, FAS, and PI3K, while activating genes that promote lipid breakdown and cholesterol conversion, such as SIRT1, PPARα, and CYP7A1.

**Figure 4:**
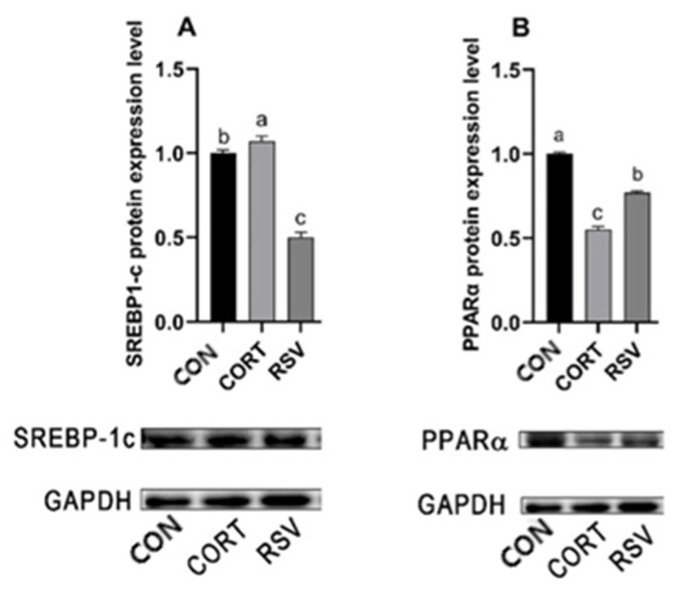
Effect of RSV on the protein levels of SREBP-1c and PPARα of broilers challenged with CORT. (**A**) SREBP-1c protein; (**B**) PPARα protein. Different letters in the same column with shoulder labels indicate significant differences (*p* < 0.05), and the same letters or no letters indicate non-significant differences (*p* > 0.05).

Figure 4 illustrates that in the CORT group, the SREBP-1c protein was significantly increased (*p* < 0.05) and the PPARα protein was significantly decreased (*p* < 0.01) in the liver of broilers compared to those in the CON group. RSV treatment significantly decreased SREBP-1c protein compared to the CORT group (*p* < 0.05). Concurrently, RSV significantly mitigated the CORT-induced reduction in PPARα protein (*p* < 0.05).

## 4. Discussion

Chronic stress, as indicated by previous research, can activate the HPA axis, leading to elevated GC secretion. This hormonal surge not only accelerates lipolysis and contributes to weight loss but also intensifies lipid deposition in the liver [11,23]. Given the liver’s vulnerability to CORT challenge, a CORT-induced chronic stress model in broiler chickens was employed to study the modulatory effects of RSV on stress responses. Our findings reveal that CORT-induced stress in AA broilers led to diminished growth performance and a pronounced rise in fat and liver indices. The livers of CORT-injected broilers exhibited severe damage, characterized by lipid metabolism disorders and aberrant fat deposition. The significant reduction in body weight might be attributed to a substantial decrease in muscle mass, thereby impairing broiler production performance. Although no significant difference in weight gain was observed in broilers supplemented with RSV, this does not imply a lack of RSV’s stress-alleviating effects. Notably, the RES group exhibited significantly lower abdominal fat and liver indices compared to the CORT group. The liver is particularly susceptible to damage following CORT injection, leading to oxidative stress, fat accumulation, and liver lipid metabolism disruption [24,25]. In this study, AA broilers administered 4 mg/kg BW of CORT via subcutaneous injection exhibited liver structural and functional impairment, characterized by hepatocyte vacuolization, hepatic edema, and varying degrees of organelle damage, including to nuclei and mitochondria. Histopathological examination revealed sections with a surface teeming with suspended lipid droplets, indicative of advanced fatty liver disease. Liu et al. [26] demonstrated that CORT induced stress in the liver tissue of Peking ducks, resulting in a significant increase in MDA content and a decrease in GSH concentration and the activities of GSH-Px, SOD, and CAT, corroborating our results, which indicated CORT’s destructive impact on the body’s antioxidant system and its role in liver cell damage. Elevated serum levels of ALT and AST are indicative of severe liver damage and diminished liver function, aligning with numerous studies that have reported liver stress effects. RSV is notable for its protective effects against various forms of liver injury [26]. Adding RSV to the feed can alleviate CORT-induced nuclear damage and atrophy in hepatocytes, enhance liver enzyme activity, improve liver structure, and reduce lipid accumulation in the liver, findings consistent with those of Karimi and Shen et al. [27,28].

Hyperlipidemia and metabolic aberrations due to endogenous GC excess are well documented in both mammals and birds [26]. There is substantial evidence that dexamethasone induces common metabolic dysfunctions and dyslipidemia, characterized by increased fasting plasma TG, TC, and LDL-C levels and decreased HDL-C levels. In mammals, elevated circulating glucocorticoids, coupled with altered insulin sensitivity, are considered significant contributors to visceral fat deposition and hyperlipidemia [29]. Exogenous glucocorticoid administration has been shown to enhance liver fat production and intramuscular lipid uptake and storage in broilers [30,31]. The liver, a pivotal organ for lipid metabolism, is prone to non-alcoholic fatty liver disease due to escalating lipid accumulation. TG and TC, the principal neutral lipids for hepatic fatty acid synthesis, when imbalanced, lead to hepatic steatosis [32]. Clinical studies have demonstrated RSV’s efficacy in improving non-alcoholic fatty liver disease [33,34]. The molecular mechanisms by which RSV alleviates hepatic steatosis include lipid metabolism regulation, reduced inflammation, mitigation of insulin resistance, and modulation of intestinal microbiota composition [35,36], with regulation of fatty acid oxidation activity and reduction in liver lipid levels as the primary therapeutic mechanisms. Studies have shown that dietary RSV supplementation reduces liver TG accumulation [34,37]. This experiment’s results indicate that dietary inclusion of RSV effectively reduced the elevated TC and TG levels and mitigated fat accumulation.

In recent years, a plethora of research has underscored the capacity of natural Nrf2 antagonists, such as vitamin C and curcumin, to mitigate liver diseases instigated by drugs, viruses, and alcohol by activating Nrf2 and modulating oxidative stress [38,39]. Nrf2, a pivotal regulator of intracellular redox equilibrium, predominantly governs in vivo antioxidant potential and is bound by its repressor Keap1 for degradation through the ubiquitin-proteasome pathway. Under oxidative stress, the adaptor protein Sqstm1/p62 interfaces with Keap1, disrupting the Nrf2–Keap1 interaction. In this study, AA broilers injected with CORT exhibited a diminished mRNA expression of Nrf2 signaling pathway-related factors and a notable reduction in hepatic Nrf2 protein expression compared to the CON group. This aligns with studies indicating that heat stress significantly reduces Nrf2 protein level in broiler muscles [40] and Nrf2 and HO-1 levels in quail liver [41]. Furthermore, our results indicate that dietary RSV supplementation effectively modulates the Nrf2 signaling pathway inhibition triggered by CORT injection.

Transcription factors such as PPARα and SREBP-1c play crucial roles in hepatic lipid metabolism. Echoing the findings of Zaytsoff et al. [12], our trial confirmed that CORT upregulates adipogenic genes, including SREBP-1c, FAS, and ACC, in avian species. RSV is an established agonist of Sirt1 and can suppress SREBP-1C-mediated hepatic lipogenesis by activating the AMPK/Sirt1 signaling pathway [42]. Our study’s findings reveal that dietary RSV supplementation suppressed lipogenic gene expression and induced PPARα gene expression. SREBP-1c, a key hepatic lipogenic transcription factor, directly engages in and regulates the transcription of lipogenic enzymes such as FAS, ACCα, and SCD. The expressional abundance of the SREBP-1c gene can influence liver fat synthesis, with elevated expression typically leading to fatty liver lesions and insulin resistance in the liver [43]. PPARα is predominantly implicated in the regulation of hepatic fatty acid oxidation; its activation facilitates the flux of fatty acids to mitochondria and peroxisomes, promoting fatty acid oxidation and reversing adipocyte differentiation [44]. Elevated hepatic PPARα expression enhances fatty acid utilization efficiency and curbs abnormal fat accumulation. Zhou et al. demonstrated that PPARα can inhibit CORT-induced visceral fat deposition in broilers [45], a finding consistent with our results. The PI3K/AKT signaling pathway is positively correlated with various obesity-related risk factors, including increased serum insulin and insulin-like growth factor levels [46]. Dietary supplementation with RSV downregulated the hepatic PI3K gene expression in AA broilers, confirming that RSV could diminish PI3K expression by upregulating Sirt1.

## 5. Conclusions

Exogenous corticosterone administration induces chronic stress in AA broilers, causing impaired growth performance, hepatic oxidative stress, excessive lipid deposition, insulin resistance, and hepatic structural and functional damage—abnormalities linked to downregulated hepatic Nrf2 antioxidant signaling and dysregulated AMPK/Sirt1-mediated lipid metabolic axis activity. Dietary supplementation with 400 mg/kg resveratrol potently ameliorates these corticosterone -induced adverse phenotypes: it activates the Nrf2 pathway to upregulate antioxidant gene expression and enhance hepatic antioxidant enzyme activity, thereby mitigating oxidative stress and preserving hepatocyte structural integrity. Concomitantly, resveratrol modulates the AMPK/Sirt1 pathway to downregulate lipogenic genes (SREBP1c, FAS, ACC, PI3K) and upregulate genes governing lipid oxidation and bile acid synthesis (PPARα, CYP7A1), normalizing serum metabolic hormone secretion, ameliorating insulin resistance, and reducing hepatic and serum triglyceride accumulation to alleviate hepatic steatosis. Resveratrol also lowers abdominal fat and liver indices and partially restores growth performance in stressed broilers, with a targeted regulatory effect on triglyceride rather than total cholesterol metabolism. Collectively, resveratrol alleviates corticosterone -induced hepatic lipid metabolic disorder and oxidative stress in AA broilers via dual modulation of the Nrf2 antioxidant pathway and AMPK/Sirt1–SREBP1c/PPARα lipid metabolic axis. This study provides a robust theoretical and practical basis for resveratrol as a natural, safe feed additive to mitigate stress-induced metabolic diseases in poultry, improve production performance and animal welfare, and reduce industry economic losses, while enriching the molecular understanding of plant extracts in regulating avian hepatic metabolism under stress.

## Figures and Tables

**Table 1:** Effect of dietary resveratrol on the growth performance, liver, and abdominal fat indexes of broilers challenged with corticosterone.

Items	Groups			F-Values	*p*-Values
	CON	CORT	RSV		
Initial BW (kg)	1.70 ± 0.01	1.70 ± 0.01	1.69 ± 0.001	0.047	0.954
Final BW (kg)	2.21 ± 0.01 ^a^	2.01 ± 0.03 ^c^	2.10 ± 0.01 ^b^	7.517	0.005
ADG (g/d) ^①^	68.21 ± 2.94 ^a^	51.79 ± 3.06 ^b^	53.57 ± 1.98 ^b^	11.092	0.001
ADFI (g/d)	139.11 ± 1.38	127.89 ± 4.76	129.48 ± 4.66	2.387	0.147
F/G	1.97 ± 0.04 ^b^	2.48 ± 0.12 ^a^	2.28 ± 0.10 ^a^	9.261	0.007
Abdominal fat index (%) ^②^	0.98 ± 0.02 ^b^	1.22 ± 0.01 ^a^	1.06 ± 0.02 ^b^	34.873	0.000
Hepatic index (%) ^③^	1.48 ± 0.02 ^c^	1.81 ± 0.03 ^a^	1.57 ± 0.02 ^b^	35.161	0.000

^a,b,c^ The means with different letters in the superscript indicate significant differences (*p* < 0.05). No broilers died during the trial. Results were expressed as mean ± SEM (n = 8). ^①^ ADG: Average daily gain, ADG = (Final BW − Initial BW)/days of feeding; ^②^ Abdominal fat index = abdominal fat weight (g)/body weight (g) × 100. ^③^ Hepatic index = liver weight (g)/body weight (g) × 100. Abbreviations: ADFI: Average Daily Feed Intake; F/G: Feed/Gain ratio.

**Table 2:** Effect of dietary resveratrol on the antioxidant status in the liver of broilers challenged with corticosterone.

Items	Groups			F-Values	*p*-Values
	CON	CORT	RSV		
T-AOC (U/mg protein)	3.56 ± 0.11 ^b^	2.68 ± 0.11 ^c^	4.31 ± 0.15 ^a^	41.648	0.000
SOD (U/mg protein)	533.96 ± 11.38 ^a^	473.27 ± 1.93 ^b^	492.00 ± 5.37 ^b^	17.836	0.000
GSH-Px (U/g protein)	30.39 ± 1.22 ^a^	24.69 ± 0.53 ^b^	28.68 ± 1.64 ^a^	8.236	0.004
CAT (U/mg protein)	37.65 ± 1.78 ^a^	29.85 ± 0.77 ^b^	35.22 ± 1.27 ^a^	8.888	0.003
MDA (nmol/mg protein)	0.38 ± 0.01 ^c^	0.54 ± 0.03 ^a^	0.45 ± 0.02 ^b^	14.911	0.000

^a,b,c^ The means with different letters in the superscript indicate significant differences (*p* < 0.05). No broilers died during the trial. Results were expressed as mean ± SEM (n = 8). Abbreviations: T-AOC: Total Antioxidant Capacity; SOD: Superoxide Dismutase; GSH-Px: Glutathione Peroxidase; CAT: Catalase; MDA: Malondialdehyde.

**Table 3:** Effect of dietary resveratrol on liver function of broilers challenged with CORT.

Items	Groups			F-Values	*p*-Values
	CON	CORT	RSV		
ALP (U/L)	2308.33 ± 115.73 ^c^	3939.67 ± 88.55 ^a^	3309.67 ± 88.36 ^b^	69.914	0.000
ALT (U/L)	3.01 ± 0.48 ^b^	6.98 ± 0.48 ^a^	3.64 ± 0.61 ^b^	17.714	0.000
AST (U/L)	284 ± 12.14 ^b^	417.35 ± 23.10 ^a^	338.59 ± 18.23 ^b^	13.302	0.000
TBA (μmol/L)	35.9 ± 1.11 ^c^	53.87 ± 1.82 ^a^	47.52 ± 0.47 ^b^	52.438	0.000

^a,b,c^ The means with different letters in the superscript indicate significant differences (*p* < 0.05). No broilers died during the trial. Results were expressed as mean ± SEM (n = 8). Abbreviations: ALP: Alkaline Phosphatase; ALT: Alanine Aminotransferase; AST: Aspartate Aminotransferase; TBA: Total Bile Acids.

**Table 4:** Effect of dietary resveratrol on hormone secretion of broilers challenged with CORT.

Items	Groups			F-Values	*p*-Values
	CON	CORT	RSV		
T3 (ng/mL)	1.08 ± 0.03 ^a^	0.94 ± 0.02 ^b^	1.07 ± 0.03 ^a^	7.944	0.004
T4 (ng/mL)	20.73 ± 0.29	20.34 ± 1.31	20.41 ± 0.34	0.066	0.937
T3/T4	0.0523 ± 0.002 ^a^	0.047 ± 0.003 ^b^	0.0523 ± 0.001 ^a^	0.122	0.015
ADP (ng/mL)	24.85 ± 0.60 ^a^	22.28 ± 0.58 ^b^	23.38 ± 0.77 ^ab^	14.354	0.001
LEP (ng/mL)	13.30 ± 0.19 ^a^	12.17 ± 0.11 ^b^	12.81 ± 0.15 ^ab^	14.034	0.000
CORT (ng/L)	407.22 ± 6.21 ^c^	474.58 ± 5.65 ^a^	455.10 ± 3.64 ^b^	43.034	0.000
INS (UIU/mL)	10.44 ± 0.23 ^b^	13.15 ± 0.42 ^a^	10.96 ± 0.27 ^b^	20.531	0.000
GLU (mmol/L)	10.91 + 0.44 ^b^	13.32 + 0.714 ^a^	11.39 + 0.41 ^b^	9.886	0.002
HOMA-IR	5.07 ± 0.25 ^b^	7.83 ± 0.623 ^a^	5.53 ± 0.13 ^b^	14.244	0.000

^a,b,c^ The means with different letters in the superscript indicate significant differences (*p* < 0.05). No broilers died during the trial. Results were expressed as mean ± SEM (n = 8). Abbreviations: T3: Triiodothyronine; T4: Thyroxine; T3/T4: Triiodothyronine/Thyroxine; ADP: Adiponectin; LEP: Leptin; CORT: Corticosterone; INS: Insulin; GLU: Glucose; HOMA-IR: Homeostatic Model Assessment for Insulin Resistance.

**Table 5:** Effect of dietary resveratrol on lipid metabolism function of broilers challenged with corticosterone.

Items	Groups			F-Values	*p*-Values
	CON	CORT	RSV		
Serum TG (mmol/L)	0.51 + 0.02 ^b^	1.14 + 0.07 ^a^	0.70 + 0.03 ^b^	47.684	0.000
Hepatic TG (mmol/L)	1.08 + 0.02 ^b^	1.75 + 0.17 ^a^	1.17 + 0.05 ^b^	10.997	0.001
Serum TC (mmol/L)	2.83 + 0.12 ^b^	4.86 + 0.13 ^a^	4.60 + 0.16 ^a^	64.354	0.000
Hepatic TC (mmol/L)	5.87 + 0.45 ^b^	10.02 + 0.15 ^a^	9.09 + 0.19 ^a^	55.060	0.000
HDL-C (mmol/L)	3.12 + 0.04 ^a^	2.91 + 0.06 ^b^	3.05 + 0.05 ^a^	8.308	0.006
LDL-C (mmol/L)	0.70 + 0.02 ^b^	0.81 + 0.03 ^a^	0.79 + 0.02 ^a^	4.699	0.026

^a,b^ The means with different letters in the superscript indicate significant differences (*p* < 0.05). No broilers died during the trial. Results were expressed as mean ± SEM (n = 8). Abbreviations: Serum TG: Serum Triglycerides; Hepatic TG: Hepatic Triglycerides; Serum TC: Serum Total Cholesterol; Hepatic TC: Hepatic Total Cholesterol; HDL-C: High-Density Lipoprotein Cholesterol; LDL-C: Low-Density Lipoprotein Cholesterol.

**Table 6:** Effect of dietary resveratrol on mRNA levels of antioxidant genes in the liver of broiler challenged with corticosterone.

Items	Groups			F-Values	*p*-Values
	CON	CORT	RSV		
Nrf2	1.00 ± 0.06 ^b^	0.53 ± 0.03 ^c^	1.84 ± 0.17 ^a^	41.800	0.000
HO-1	1.00 ± 0.08 ^b^	0.74 ± 0.60 ^c^	1.70 ± 0.10 ^a^	39.679	0.000
CAT	1.00 ± 0.02 ^b^	0.65 ± 0.02 ^b^	1.62 ± 0.27 ^a^	9.993	0.002
SOD	1.00 ± 0.14 ^b^	0.66 ± 0.04 ^b^	1.41 ± 0.15 ^a^	9.573	0.002
NQO1	1.00 ± 0.15 ^b^	1.03 ± 0.10 ^b^	1.40 ± 0.13 ^b^	2.324	0.132
GSH-Px	1.00 ± 0.05 ^b^	0.62 ± 0.11 ^c^	1.42 ± 0.13 ^a^	15.367	0.000

^a,b,c^ The means with different letters in the superscript indicate significant differences (*p* < 0.05). No broilers died during the trial. Results were expressed as mean ± SEM (n = 8). Abbreviations: Nrf2: Nuclear factor erythroid 2-related factor 2; HO-1: Heme oxygenase-1; CAT: Catalase; SOD: Superoxide dismutase; NQO1: NAD (P)H: quinone oxidoreductase 1; GSH-Px: Glutathione peroxidase.

**Table 7:** Effect of dietary resveratrol on mRNA levels of lipid-metabolism genes in broilers challenged with corticosterone.

Items	Groups			F-Values	*p*-Values
	CON	CORT	RSV		
SIRT1	1.00 ± 0.12 ^b^	0.89 ± 0.13 ^b^	2.64 ± 0.17 ^a^	46.652	0.000
SREBP1c	1.00 ± 0.13 ^b^	3.00 ± 0.24 ^a^	2.37 ± 0.24 ^a^	23.900	0.000
FAS	1.00 ± 0.04 ^c^	2.21 ± 0.18 ^a^	1.41 ± 0.13 ^b^	22.303	0.000
ACC	1.00 ± 0.15 ^c^	3.61 ± 0.16 ^a^	1.81 ± 0.17 ^b^	72.303	0.000
PPARα	1.00 ± 0.07 ^b^	1.01 ± 0.04 ^b^	1.35 ± 0.09 ^a^	8.393	0.004
CYP7A1	1.00 ± 0.13 ^b^	1.04 ± 0.04 ^b^	3.15 ± 0.11 ^a^	143.299	0.000
PI3K	1.00 ± 0.09 ^b^	1.60 ± 0.09 ^a^	1.13 ± 0.10 ^b^	11.012	0.001

^a,b,c^ The means with different letters in the superscript indicate significant differences (*p* < 0.05). No broilers died during the trial. Results were expressed as mean ± SEM (n = 8). Abbreviations: SIRT1: Sirtuin 1; SREBP1c: Sterol Regulatory Element-Binding Protein-1c; FAS: Fatty Acid Synthase; ACC: Acetyl-CoA Carboxylase; PPARα: Peroxisome Proliferator-Activated Receptor αlpha; CYP7A1: Cytochrome P450 Family 7 Subfamily A Member 1; PI3K: Phosphatidylinositol 3-Kinase.

## Data Availability

The data that support the findings of this study are available from the corresponding author upon reasonable request.

## Supplementary Materials

The following supporting information can be downloaded at https://www.mdpi.com/article/10.3390/ani16111574/s1. Table S1: Composition and nutrition levels of the basal diet; Table S2: Primers used for Real-time PCR.
