# Supplementary material for: Resveratrol Alleviates Corticosterone-Induced Hepatic Lipid Metabolism Disorder and Oxidative Stress by Regulating the Nrf2 and AMPK/Sirt1 Signaling Pathways in AA Broilers
Source: Animals (Basel). 2026 May 22;16(11):1574. doi: 10.3390/ani16111574 (PMC13255576; doi:10.3390/ani16111574)
Supplement: Supplementary file 1 [file animals-16-01574-s001.zip › animals-4281672-supplementary.pdf]

SUPPLEMENTARY TABLES

Supplementary Table S1. Composition and nutrition levels of the basal diet.

Ingredients (%)	0-21 days	22-42 days	Nutrient levels [2]	0-21 days	22-42 days
Corn	58.50	61.15	Digestible energy (MJ/kg)	12.54	12.96
Soybean meal	30.0	26.3	Crude protein (%)	21.50	20.09
Soybean oil	2.70	3.80	Lysine (%)	1.15	1.01
Corn gluten meal	4.06	4.33	Methionine (%)	0.55	0.43
Methionine	0.21	0.10	Methionine + Cysteine (%)	0.91	0.77
L-Lysine	0.20	0.14	Threonine (%)	0.80	0.73
Dicalcium phosphate	1.60	1.52	Calcium (%)	1.06	0.91
Limestone	1.33	1.26	Total phosphorus (%)	0.73	0.69
Sodium chloride	0.30	0.30	Available phosphorus (%)	0.45	0.43
Choline chloride	0.10	0.10			
Premix[1]	1.00	1.00			
Total	100.00	100.00			

Provided the following per kilogram of diet: vitamin A, 12000 IU; vitamin D3, 2500 IU; vitamin E, 20 IU; vitamin K3, 1.3 mg; thiamine, 2.2 mg; riboflavin, 8.0 mg; nicotinamide, 40 mg; calcium pantothenate, 10 mg; pyridoxine, 4 mg; biotin, 0.04 mg; folic acid, 1 mg; vitamin B_{12}, 0.013 mg; Fe (from ferrous sulfate), 80 mg; Cu (from copper sulfate), 8.0 mg; Mn (from manganese sulfate), 110 mg; Zn (from zinc sulfate), 60 mg; I (from calcium iodate), 1.1 mg; Se (from sodium selenite), 0.3 mg. Data were calculated values.

Supplementary Table S2. Primers used for Real-time PCR.

Genes	Sequence (5′ to 3′)	Product size (bp)	GenBank No.
β-actin	F: TGCGTGACATCAAGGAGAAG<br>R: TGCCAGGGTACATTGTGGTA	300	NM_205518.2
Sirt1	F: GATCAGCAAAAGGCTGGATGGT<br>R: ACGAGCCGCTTTCGCTACTAC	143	NM_001004767.2
AMPK	F: GGGACCTGAAACCAGAGAACG<br>R: ACAGAGGAGGGCATAGAGGATG	215	NM_001039605.2
SREBF1	F: CTACCGCTCATCCATCAACG<br>R: CTGCTTCAGCTTCTGGTTGC	145	NM_204126.3
SCD	F: GCCTTCCAGAATGACATCTATG<br>R: AAATCACTCAGGTCCAGCTTC	182	NM_204890.2
ACC	F: GCCTCCGAGAACCCAA<br>R: CCAGCAGTCTGAGCCACTA	128	NM_205505.2
FASN	F: TCTCTGCCATCTCCCGAACTTCC<br>R: TCTCAATTAGCCACTGTGCCAACTC	102	NM_205155.4
CYP7A1	F: GTAACGCCCTAGATGCCCTC<br>R: GCTCTCTCTGTTTCCCGCTT	220	NM_001001753.2
PPARα	F: CCTTTCACCAGCATCCAGTCCTTC<br>R: TGTACTCCGTAATGGTAGCCTGAGG	138	NM_001001464.1
NRF2	F: CGCTTTCTTCAGGGGTAGCA<br>R: AGTTCGGTGCAGAAGAGGTG	170	NM_205117.2
CAT	F: GGTTCGGTGGGGTTGTCTTT<br>R: CACCAGTGGTCAAGGCATCT	213	NM_001031215.2
HO-1	F: ACGAGTTCAAGCTGGTCACG<br>R: GGATGCTTCTTGCCAACGAC	244	NM_205344.2
GSH-PX	F: TGGGGAATGCCATCAAGTGG<br>R: TCCTCCATTGGGCTGTACCT	85	NM_204220.3
NQO1	F: ACCTCTTTCAACCACGCCAT<br>R: GAGATGGTGACTTCCCAGCC	74	NM_001277620.2
SOD	F: CCGGCTTGTCTGATGGAGAT<br>R: TGCATCTTTTGGTCCACCGT	125	NM_205064.2
PI3K	F: CGGATGTTGCCTTACGGTTGT<br>R: GTTCTTGTCCTTGAGCCACTGAT	162	NM_001004410.2
AKT	F: GGCTCCACAAACGAGGAGAA<br>R: GGCTGTCTGCAACAGTTTGG	297	NM_001396387.1
